# Modulation of some markers of erectile dysfunction and malonaldehyde levels in isolated rat penile tissue with unripe and ripe plantain peels: identification of the constituents of the plants using HPLC

**DOI:** 10.1080/13880209.2017.1340966

**Published:** 2017-06-27

**Authors:** Ganiyu Oboh, Adedayo Oluwaseun Ademiluyi, Sunday Idowu Oyeleye, Tosin Abiola Olasehinde, Aline Augusti Boligon

**Affiliations:** a Department of Biochemistry, Federal University of Technology, Akure, Nigeria;; b Department of Biomedical Technology, Federal University of Technology, Akure, Nigeria;; c Division of Nutrition and Toxicology, Department of Food Technology, Federal Institute of Industrial Research Oshodi, Lagos, Nigeria;; d Phytochemical Research Laboratory, Department of Industrial Pharmacy, Federal University of Santa Maria, Santa Maria, Brazil

**Keywords:** phosphodiesterase-5, arginase, acetylcholinesterase, angiotensin-I converting enzyme, lipid peroxidation, antioxidant, polyphenols

## Abstract

**Context:** Plantain fruit pulp has been used as a natural remedy to manage erectile dysfunction (ED) in traditional medicine. However, the potency of the peel has not been examined with respect to ED management.

**Objective:** This study investigated and compared the inhibitory potential of unripe (UPP) and ripe (RPP) plantain peels on some enzymes associated with ED and Fe^2+^-induced oxidative stress in albino rat penile homogenate *in vitro*.

**Materials and method:** Aqueous extract of the peels was prepared and the effect on phosphodiesterase-5 (PDE-5), arginase, acetylcholinesterase (AChE), angiotensin-I converting enzyme (ACE) and Fe^2+^-induced malonyladehyde in isolated albino rat penile homogenate were investigated. Phenolic constituents of the peels powder were characterized using high-performance liquid chromatography coupled with diode array detector (HPLC-DAD).

**Result:** Extract from UPP had higher PDE-5 (IC_50_ = 3.10 μg/mL), arginase (IC_50_ = 0.96 μg/mL), AChE (IC_50_ = 6.30 μg/mL) and ACE (IC_50_ = 0.41 μg/mL) inhibitory ability compared with RPP (PDE-5, IC_50_ = 4.33 μg/mL; arginase, IC_50_ = 1.34 μg/mL; AChE, IC_50_ = 8.64 μg/mL; ACE, IC_50_ = 0.63 μg/mL). The extract from UPP also had higher inhibition of Fe^2+^-induced lipid peroxidation. HPLC-DAD analysis revealed that gallic and caffeic acids, rutin, quercitrin and quercetin were abundant in UPP, while catechin, kaempferol, chlorogenic and ellagic acids were the dominant phenolic compounds in RPP.

**Discussion and conclusion:** Inhibition of enzymes associated with ED and lipid peroxidation could be linked with the phenolic compounds. However, UPP appeared to be more potent.

## Introduction

Erectile dysfunction (ED) is a common form of sexual dysfunction that is rampant among sexually active men, and it is estimated to affect about 322 million men globally by the year 2025 (Ismail and El-Sakka 2016). Kandeel et al. (2001) reported that ED is a vascular disorder that is characterized by difficulty and/or inability to attain/maintain penile erection for vaginal penetration. Reports have revealed that the process of penile erection involve multiple factors which include vascular, hormonal, psychological and neurological factors and any alteration affecting the aforementioned factors may induce ED (Kandeel et al. 2001; Guay et al. 2003).

Research has shown that cyclic-guanine monophosphate (cGMP), nitric oxide (NO) and acetylcholine (ACh) play an active role in the erection process (Kandeel et al. 2001; Corbin 2004). Hence, low levels of these cellular messengers could lead to impaired erection. For instance, the up-regulation of phosphodiesterase type 5 (PDE-5) reduces the levels of cGMP in penile tissue, thus impair penile erection (Boswell-Smith et al. 2006). In addition, the increased level of cGMP in penile tissues is dependent on NO-induced activation of guanyl cyclase, hence, maximum concentration of NO in the penile tissue is required (Cerqueira et al. 2008). In the ED, the increased activity of arginase reduces production of NO, as arginase catalyzes the conversion of arginine to urea and ornithine, thereby reducing arginine levels that could be used for NO production by NO synthase (NOs). Arginase competes with NOs for l-arginine substrate, therefore, inhibition of arginase could up-regulate NO production (Sakai et al. 2004; Oboh et al. 2015). Inhibition of acetylcholinesterase (AChE) is another target for the treatment of ED as it regulates the levels of ACh, which trigger NO-dependent smooth muscle relaxation in erection process (Vargas et al. 2004). The penile tissue is a reservoir of cholinergic nerves and ACh molecule (Hedlund et al. 2000). But, the increase activity of AChE reduces the concentration of ACh by catalyzing the conversion of acetylcholine to acetate and choline, respectively (Akomolafe et al. 2016). As reported, inhibition of AChE increases the level of ACh, and consequently improves penile erection and rigidity (Andersson 2011; Nunes and Webb 2012). Furthermore, the rennin–angiotensin system (RAS) is not only a major factor in the pathophysiology of hypertension but also in ED. The increase in the production of angiotensin-II as a result of elevated angiotensin-I converting enzyme (ACE) activity induces ED (Bader 2010; Fraga-Silva et al. 2013; Oboh et al. 2015; Akomolafe et al. 2016). Therefore, the inhibition of ACE activity in turn reduces angiotensin-II levels and improves erectile function in ED patients (Dorrance et al. 2002).

Several studies have reported the relationship between ED and oxidative stress (Oboh et al. 2015; Akomolafe et al. 2016). The reaction between superoxide and NO forms a highly toxic radical such as peroxynitrite, which is capable of reducing the available NO required for penile erection (Khan et al. 2001; Agarwal et al. 2006). The use of antioxidants has been reported to ameliorate oxidative stress via scavenging of free radicals and chelation of transition metals (Adefegha et al. 2015; Ademiluyi et al. 2015). So far, polyphenols which are abundant in plant-based human diet such as fruits and vegetables have been reported to be efficient in the management of ED and other related diseases such as hypertension with little or no side effects (Ferreira et al. 2006; Oboh et al. 2015; Akomolafe et al. 2016).

The management of ED with orthodox drugs is associated with diverse adverse side effects. This has motivated a continuous search for herbal/medicinal plant with erectogenic potentials as there are less side effects associated with herbal medications (Saxena et al. 2012; Oboh et al. 2015; Akomolafe et al. 2016). Plantain [*Musa sapientum* Linn. var. *paradisiaca* Juss.] is a tropical fruit and staple food crop with over 2.11 million metric tons produced in Nigeria annually. The pulp is being used in traditional medicine for the management of ED. However, the potency of the peel has not been examined with respect to the management of ED. Hence, to the best of our knowledge, there is a dearth of information on whether the peels which makes up to 40% of the total weight and constitutes a waste problem could exert biological effects towards the management of ED. In this study, the effects of aqueous extracts from unripe (UPP) and ripe (RPP) plantain peels on some enzymes (PDE-5, arginase, AChE and ACE) associated with ED and malonyladehyde levels in isolated penile homogenate of rats were investigated. Antioxidant activity [ferric reducing antioxidant property (FRAP)] of the peels were also quantified and their phenolic constituents were further characterized using high-performance liquid chromatography (HPLC) coupled with diode array detector (DAD).

## Materials and methods

Chemicals were of analytical grade. Methanol, formic acid, gallic acid (purity ≥95% HPLC), caffeic acid (purity ≥95% HPLC), chlorogenic acid (purity ≥95% HPLC) and ellagic acid (purity ≥98% HPLC) were purchased from Merck (Darmstadt, Germany). Catechin (purity ≥98% HPLC), epicatechin (purity ≥95% HPLC), quercetin (purity ≥96% HPLC), quercitrin (purity ≥96% HPLC), rutin (purity ≥96% HPLC) and kaempferol (purity ≥95% HPLC) were acquired from Sigma Chemical Co. (St. Louis, MO). High-performance liquid chromatography (HPLC-DAD) was performed with a Shimadzu Prominence Auto Sampler (SIL-20A) HPLC system (Shimadzu, Kyoto, Japan), equipped with Shimadzu LC-20AT reciprocating pumps connected to a DGU 20A5 degasser with a CBM 20A Integrator, SPD-M20A diode array detector and LC solution 1.22 SP1 software (SPSS Inc., Chicago, IL). Kenxin refrigerated centrifuge Model KX3400C and UV–visible spectrophotometer (Model 6305; Jenway, Barloworld Scientific, Dunmow, United Kingdom) were used for centrifuging and the measurement of the absorbance respectively. Distilled water (DW) was used for the preparation of reagents.

## Sample collection and preparation of extracts

Mature unripped (UPP) and ripped (RPP) plantain fruits were purchased from Akure main market (locally known as Oja-Oba market in Southwest Nigeria; 7.2500°N, 5.1950°E), Akure, and was identified and authenticated by Mr. A. A. Shorungbe in Biology Department, Federal University of Technology, Akure, Nigeria. The voucher number (FUTA/BIO/302) was deposited in the University herbarium. The fruits were thoroughly washed, thereafter the pulps were carefully removed using table knife, the peels were dried and pulverized, using laboratory blender, and sieved in Willey 60 mesh sizes. A hundred gram of each sample was weighed and extracted with 500 mL of distilled water. The filtrate was freeze dried, thereafter each dried extract (100 mg) was reconstituted with 100 mL of distilled water and stored in the refrigerator for subsequent analysis. The %yield was calculated based on the amount of freeze dried extract (g) obtained from 100 g of the peel DW multiply by 100. The freeze dried samples were used for HPLC-DAD analysis.

## Handling of experimental animals

Forty adult male Wistar strain albino rats weighing 280–290 g were procured from the animal colony of Animal Production and Health Department, Federal University of Technology, Akure, and were handled according to the guide for the Care and Use of Laboratory Animals, published by the National Institute of Health (NIH), USA. The ethical guideline was followed in accordance with National and Institutional guidelines for the protection of animal welfare during the experiments (reference number FUTA/SOS/1411). The rats were maintained at 25.0 ± 2 °C on a 12 h light/dark cycle with access to standard animal feed and water *ad libitum* for 15 d before the commencement of the experiment.

## Inhibition of phosphodiesterase-5 (PDE-5) activity assay

The penile tissue was carefully removed and homogenized with three volumes of ice-cold buffer [0.1 M Tris-HCl buffer containing 1 mM CaCl_2_ and 50 mM NaCl (pH 8.0)]. The ability of the extracts to inhibit PDE-5 activity was assessed (Oboh et al. 2017b). The reaction mixture containing 5 mM of the substrate (*p*-nitrophenyl phenylphosphonate), tissue homogenate, 20 mM Tris buffer (pH 8.0) and the extracts/sildenafil were incubated at 37 °C for 10 min. The intensity of *p*-nitrophenol produced was measured as a change in absorbance after 5 min at 400 nm. The control experiment was performed without the extracts/sildenafil. The PDE-5 inhibitory activity was expressed as percentage inhibition using the formula below:

(1) $$\text{PDE-5}\text{inhibition }\left(\%\right)=\left[\left({\text{Abs}}_{\text{control}}–{\text{ Abs}}_{\text{samples}}\right)/{\text{Abs}}_{\text{control}}\right]\times \text{1}00$$

where Abs_control_ is the absorbance without the extract and Abs_samples_ is the absorbance with extract.

## Inhibition of arginase activity assay

Penile tissue was homogenized with three volumes cold buffer (phosphate buffer, pH 7.2), and centrifuged for 20 min at 357.80*g*. The supernatant was used as a source of arginase, in which the activity was determined using Adefegha et al. (2015) method in a reaction mixture containing Tris–HCl buffer (1.0 mM, pH 9.5, 1.0 mM MnCl), 0.1 M arginine solution and extract/l-2-amino-[4-(2′-hydroxyguanidino)] butyric acid (l-NOHA). The mixture was made to a final volume of 1.0 mL. The mixture was incubated for 10 min at 37 °C. The reaction was terminated by the addition of 2.5 mL Ehrlich reagent [2.0 g of *p*-dimethylaminobenzaldehyde in 20 mL of absolute hydrochloric acid (37% purity) and made up to 100 mL with distilled water]. The absorbance was read after 20 min at 450 nm. The control experiment was performed without the test sample or standard and arginase inhibitory activity was calculated [Equation (1)] and expressed as %inhibition [Equation (1)].

## AChE inhibitory assay

Homogenate of the rat penile tissue was prepared in three volumes of cold buffer (phosphate buffer, 0.1 M, pH 7.2) and used as the source of AChE (EC 3.1.1.7). The effect of the extracts/prostigmine on AChE activity was assessed using colorimetric method (Akomolafe et al. 2016). The AChE activity was determined in a reaction containing of 200 μL tissue homogenate, 100 μL of 5,5′-dithio-bis(2-nitrobenzoic) acid (DTNB 3.3 mM), extracts or lisinopril and phosphate buffer, pH 8.0. The mixture was incubated for 20 min at 25 °C, and the substrate (acetylthiocholine iodide) was added. Immediately, the enzyme activity was measured at 412 nm. The AChE activity was thereafter expressed as %inhibition using Equation (1).

## Angiotensin-I converting enzyme (ACE) inhibitory assay

The effect of the extracts on ACE activity using the method of Akomolafe et al. (2016) was investigated. The extracts/lisinopril and 50 μL of the penile homogenate as a source for ACE (EC 3.4.15.1) were preincubated at 37 °C for 15 min. The ACE substrate [150 μL, 8.33 mM hippuryl-l-histidyl-leucine in 125 mM of Tris–HCl buffer (pH 8.3)] was added to the mixture which was incubated at 37 °C for 30 min. The reaction was hallted with 250 μL of 1 M HCl. The enzymatic product [hippuric acid (Bz-Gly)] was extracted with 1.5 mL of ethyl acetate, and centrifuged to separate the ethyl acetate layer. Thereafter, 1 mL of ethyl acetate layer was transferred to a clean test tube and evaporated to dryness. Distilled water (1 mL) was added and its absorbance was measured at 228 nm. The control experiment was performed without the test sample/lisinopryl. The percentage ACE inhibition was subsequently calculated (Equation (1)).

## Preparation of penile homogenate and lipid peroxidation assay

Penile homogenate was prepared according to the method of Belle et al. (2004) and lipid peroxidation assay was determined using the method of Ademosun et al. (2016). The rat penile homogenate (100 μL) was mixed with a mixture containing 30 μL of 0.1 M Tris-HCl buffer (pH 7.4), extract and 30 μL of 250 μM Fe^2+^. The volume was made up to 300 μL with distilled water, and incubated at 37 °C for 2 h. The colour reaction was developed by adding 300 μL of 8.1% sodium dodecyl sulphate, 600 μL of acetic acid/HCl (pH 3.4) and 600 μL of 0.8% thiobarbituric acid. The thiobarbituric acid reactive species (TBARS) produced was measured at 532 nm and subsequently calculated as the percent of malondialdehyde (MDA) produced (% control) using the MDA standard curve.

## FRAP assay

The ability of the extract to reduce FeCl_3_ solution, using ascorbic acid as a standard, was determined by mixing 500 μL of the extract with 2.5 mL of 200 nM of phosphate buffer (pH 6.6) and 2.5 mL of 1% potassium ferricyanide. The mixture was incubated for 20 min at 50 °C, followed by the addition of 2.5 mL of 10% trichloroacetic acid. After centrifugation at 650 rpm for 10 min, 5 mL of the supernatant was mixed with an equal volume of distilled water and 1 mL of 0.1% ferric chloride. The absorbance was measured at 700 nm and ferric reducing ability of the extract was calculated and expressed as ascorbic acid equivalent (AAE) (Oyaizu 1986).

## Spectrophotometric determination of total phenol and flavonoid contents

The total phenol content of the extract using gallic acid as standard was determined by oxidized with 2.5 mL 10% Folin–Ciocalteu’s reagent (v/v) and neutralized by 2.0 mL of 7.5% Na_2_CO_3_. The mixture was incubated at 45 °C for 40 min, and the absorbance was measured at 765 nm. The total phenol content was subsequently calculated as gallic acid equivalent (GAE). The total flavonoid content of the extracts was also determined in a reaction mixture containing 0.5 mL of the extract and methanol, 50 μL of 10% AlCl3, 50 μL of 1 M potassium acetate and 1.4 mL of distilled water. The mixture was incubated at 25 °C for 30 min. The absorbance was subsequently measured at 415 nm. Quercetin was used as a standard and the total flavonoid content was calculated as quercetin equivalent (QE) (Akomolafe et al., 2016).

## Quantification of phenolic compounds by HPLC-DAD

Reverse phase chromatography analyses were carried out under gradient conditions using a C_18_ column (4.6 mm × 150 mm) packed with 5 μm diameter particles; the mobile phase was water containing 1% formic acid (A) and acetonitrile (B), and the composition gradient was 13% of B until 10 min and changed to obtain 20, 30, 50, 60, 70, 20 and 10% B in 20, 30, 40, 50, 60, 70 and 80 min, respectively, following the method described by Adedayo et al. (2015) with slight modifications. Plantain peel extracts and mobile phase were filtered through membrane filter (0.45 μm Millipore, Billerica, MA) and then degassed by ultrasonic bath prior to use, the extracts were analyzed at a concentration of 15 mg/mL. The flow rate was 0.8 mL/min, injection volume of 40 μL and the wavelength were 254 for gallic acid, 280 for catechin and epicatechin, 325 nm for chlorogenic, caffeic and ellagic acids, and 365 nm for quercetin, quercitrin, rutin and kaempferol. All the samples and the mobile phase were filtered through a 0.45-μm membrane filter (Millipore, Billerica, MA) and then degassed by ultrasonic bath prior to use. Stock solutions of standard references were prepared in the mobile phase at a concentration range of 0.030–0.250 mg/mL for kaempferol, quercetin, quercitrin, isoquercitrin, rutin, catechin and epicatechin; and 0.050–0.450 mg/mL for ellagic, gallic and chlorogenic acids. Chromatography peaks were confirmed by comparing its retention time with those of reference standards and by DAD spectra (200–500 nm). Calibration curve for gallic acid: *Y* = 12,618*x* + 1196.2 (*r* = 0.9996); chlorogenic acid: *Y* = 11,953*x* + 1367.5 (*r* = 0.9998); caffeic acid: *Y* = 12,736*x* + 1365.1 (*r* = 0.9993); ellagic acid: *Y* = 12,743*x* + 1246.3 (*r* = 0.9991); catechin: *Y* = 13,089*x* + 1257.9 (*r* = 0.9995); epicatechin: *Y* = 12,649*x* + 1189.1 (*r* = 0.9995); quercitrin: *Y* = 13,165*x* + 1205.7 (*r* = 0.9999); rutin: *Y* = 13,983*x* + 1171.3 (*r* = 0.9998); quercetin: *Y* = 13,165*x* + 1292.5 (*r* = 0.9996) and kaempferol: *Y* = 12,539*x* + 1183.0 (*r* = 0.9997). All chromatography operations were carried out in triplicate at ambient temperature. The limit of detection (LOD) and limit of quantification (LOQ) were calculated based on the standard deviation of the responses and the slope using three independent analytical curves. LOD and LOQ were calculated as 3.3 and 10 *σ*/*S*, respectively, where *σ* is the standard deviation of the response and *S* is the slope of the calibration curve (Akomolafe et al. 2016).

## Data analysis

The results of three replicate experiments were pooled and expressed as mean ± standard deviation (SD). Mean values were analyzed and compared using Student’s *t* test (unpaired). The significance was accepted at a *p* value ≤0.05. The extract concentration causing 50% antioxidant/enzyme activities (IC_50_) value was determined using non-linear regression analysis with Graph Pad Prism version 5.00 for Windows (GraphPad Inc., San Diego, CA).

## Results

The PDE-5 inhibitory potential of the UPP and RPP extracts and sildenafil were investigated and the IC_50_ values (lower IC_50_ value means stronger enzyme inhibition) are listed in Table 1. The result revealed that UPP had higher PDE-5 inhibitory activity compared with RPP. However, sildenafil had the highest. Effects of the UPP and RPP extracts as well as l-NOHA on arginase activity in isolated rat’s penile homogenate were also investigated. The result showed that the extracts inhibited arginase activity in a concentration-dependent manner, as revealed by the IC_50_ values listed in Table 1. l-NOHA had the highest, followed by UPP, while RPP had the least arginase inhibitory potential. The result of the AChE inhibitory potential of the extracts and standard drug (Prostigmine) showed that UPP extract had highest inhibitory effect compared with RPP extract (Table 1), while prostigmine had the highest. The interaction of the studied peel extracts and lisinopril on ACE revealed that the extracts inhibited ACE activity in a concentration-dependent manner. UPP extract exhibited higher inhibitory effect compared with RPP, while lisinopril had the highest ACE inhibitory potential (Table 1).

**Table 1.** IC_50_ values (μg/mL) of PDE-5, arginase, ACE and AChE inhibitory activities in rat penile tissue by unripe (UPP) and ripe (RPP) plantain peel extracts and standard drugs/inhibitors.

Sample	PDE-5	Arginase	AChE	ACE
UPP	3.10 ± 0.03^b^	0.96 ± 0.04^b^	6.30 ± 0.03^b^	0.41 ± 0.04^b^
RPP	4.33 ± 0.06^c^	1.34 ± 0.09^c^	8.64 ± 0.01^c^	0.63 ± 0.03^c^
Sildenafil*	2.68 ± 0.10^a^	–	–	–
L-NOHA*	–	0.78 ± 0.20^a^	–	–
Prostigmine*	–	–	2.35 ± 0.22^a^	–
Lisinopril*	–	–	–	0.20 ± 0.05^a^

Values represent mean ± standard deviation (*n* = 3). Values with the same superscript along the column are not significant (*p* < .05) different. Sildenafil*: standard drug for PDE-5; L-NOHA*: standard inhibitor for arginase; prostigmine*: standard drugs for AChE; lisinopril*: standard drug for ACE.

**Figure 1.**
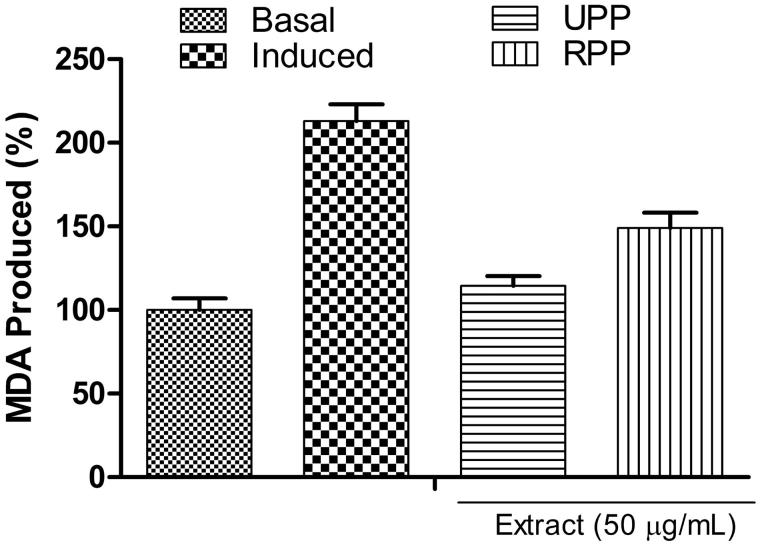
Inhibition of Fe^2+^-induced lipid peroxidation in rat penis by aqueous extracts of unripe (UPP) and ripe (RPP) plantain peels.

Figure 1 depicts the effect of the extracts on Fe^2+^-induced lipid peroxidation in rat’s penile homogenate. The incubation of Fe^2+^ solution with penile homogenate induced lipid peroxidation (213.01%) as against the basal (100%). However, the addition of the extracts inhibited lipid peroxidation, and UPP extract (114.41%) had the highest inhibitory effect relative to RPP (149.15%). In addition, the reducing power of the extracts reported as AAE (Table 2) revealed that both extracts were able to reduce Fe (III) to Fe (II); however, UPP had higher reducing power compared with RPP.

**Table 2.** Percentage (%) yield, total phenol (GAE), total flavonoid (QE) and FRAP (AEE) contents of aqueous extracts from unripe (UPP) and ripe (RPP) plantain peels (mg/100 g).

Sample	% yield	Total phenol	Total flavonoid	FRAP
UPP	13.20	337.5 ± 1.9^a^	187.5 ± 2.1^a^	365.8 ± 1.1^a^
RPP	11.01	233.8 ± 2.2^b^	110.6 ± 1.7^b^	235.8 ± 2.4^b^

Values represent means of triplicate. Values with the same alphabet along the same column are not significantly different (*p* > 0.05).

**Figure 2.**
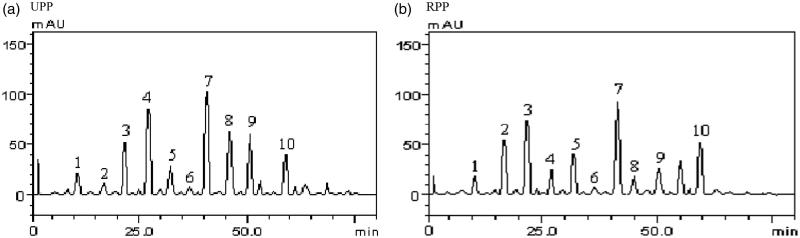
HPLC chromatograms of UPP and RPP. Peaks 1–10 represent gallic acid, catechin, chlorogenic acid, caffeic acid, ellagic acid, epicatechin, rutin, quercitrin, quercetin and kaempferol.

The %yield of the extract is presented in Table 2. The distribution of the phenol and flavonoid contents of the extracts reported as the GAE and QE, respectively, is presented in Table 2. UPP had the highest total phenol and flavonoid contents than RPP. The HPLC phenolic profile of the extracts shown in Table 3 and Figure 2 revealed the abundance of individual components. Gallic and caffeic acids, epicatechin, rutin and quercitrin quercetin were relatively higher in UPP compared with RPP. However, RPP had higher levels of chlorogenic acid, epicatechin, ellagic acid and kaempferol.

**Table 3.** Phenolics composition of unripe (UPP) and ripe (RPP) *Plantain* peel extracts.

	Plantain peel		LOD	LOQ
Compounds	UPP (mg/g)	RPP (mg/g)	μg/mL	μg/mL
Gallic acid	6.07 ± 0.01^a^	5.64 ± 0.02^a^	0.007	0.023
Catechin	3.89 ± 0.03^b^	21.38 ± 0.01^b^	0.025	0.082
Chlorogenic acid	19.74 ± 0.01^c^	28.13 ± 0.03^c^	0.018	0.059
Caffeic acid	30.15 ± 0.02^d^	8.79 ± 0.01^d^	0.015	0.049
Ellagic acid	8.69 ± 0.03^e^	16.08 ± 0.01^e^	0.031	0.102
Epicatechin	3.87 ± 0.01^b^	3.75 ± 0.02^f^	0.016	0.059
Rutin	35.18 ± 0.01^f^	32.41 ± 0.03^b^	0.024	0.081
Quercitrin	23.45 ± 0.02^g^	5.02 ± 0.02^a^	0.009	0.027
Quercetin	23.07 ± 0.01^g^	8.93 ± 0.01^d^	0.013	0.042
Kaempferol	17.02 ± 0.01^h^	20.68 ± 0.02^b^	0.032	0.037

Results are expressed as mean ± standard deviations (SD) of three determinations. Averages followed by different letters differ by Tukey test at *p* < 0.01.

## Discussion

Inhibition of PDE-5 is pivotal to the penile erectile process. PDE-5 catalyzes the breakdown of the cGMP and also reduces NO levels in the endothelial cells, thereby decreasing signalling (Boswell-Smith et al. 2006). The use of PDE-5 inhibitors such as sildenafil, tadalafil and verdanafil does not only raise the levels of cGMP (Chuang et al. 1998; Corbin 2004) but also stimulates activation of cGMP and increases NO bioavailability for the relaxation of penile tissue that results in penile erection. However, these inhibitors also elicit several side effects, including headache, dyspepsia, nasal congestion, visual abnormalities among others (Saxena et al. 2012). The result revealed that PDE-5 activity was inhibited by the extracts concentration dependently. This finding is in line with previous reports that medicinal plant extracts can inhibit PDE-5 activity, especially plants rich in flavonoids (Dell'Agli et al. 2008; Shin et al. 2015; Kotirum et al. 2015; Pavan et al. 2015). The PDE-5 inhibitory potentials of the studied peels could be linked to their rich flavonoid.

The utilization of l-arginine by arginase activity in vascular endothelia and smooth muscle cells of penile tissue can reduce NOS activity and consequently decrease concentration of NO: a major factor in erectile function (Mori and Gotoh 2000). Hence, the inhibition of arginase activity can be additional therapeutic targets for the management of ED as this could increase the bioavailability of l-arginine, contribute to the production of NO via reaction catalyzed by NOS, where overall result could facilitate penile erection. Inhibition of arginase activity by the studied extracts is in line with some reports of plant extracts, which are rich in phenolics including epicatechin, quercetin, quercitrin and isoquercitrin and their arginase inhibitory potential (Shin et al. 2011; Oboh et al. 2015; Akomolafe et al. 2016). Hence, the observed high flavonoid content and abundant presence of flavonoid compounds such as (–)-epicatechin, rutin, quercetin and quercitrin in UPP may be responsible for its higher arginase inhibitory activity. Nevertheless, both sample extracts have better arginase inhibitory ability than that of *Moringa oleifera* Lam (Moringaceae) (Oboh et al. 2015) and *Ficus capensis* Thunb (Moraceae) leaves (Akomolafe et al. 2016).

Several studies have reported that the penile tissue is rich in cholinergic nerves, and for sexual stimulation to occur, the nerves must be able to release ACh for the stimulation of NO production from l-arginine by the catalytic action of neuronal nitric oxide synthase (nNOS) (Hedlund et al. 2000; Vargas et al. 2004). The inhibition of AChE by the extracts substantiate the folkloric use of plantain peels in the management and/or treatment of ED. The inhibition of AChE by the peels correlates with recent studies that plant derived AChE inhibitors can be employed in ameliorating ED, and can also prevent oxidative stress in neuronal cells of penile tissue (Ramassamy 2006; Hostettmann et al. 2006; Butterfield and Lauderback 2002; Andersson 2011; Nunes and Webb 2012; Akomolafe et al. 2016). The inhibition of AChE by the extracts could be associated with the presence of phenolic compounds. This agrees with recent studies that phenolic-rich plants could inhibit AChE (Akomolafe et al. 2016; Nwanna et al. 2016; Oboh et al. 2017a). This also confirms the fact that UPP which had the highest phenolics possessed the highest AChE inhibitory potentials.

Persistence increase in blood pressure, otherwise known as hypertension, has been reported as one of the risk factors of ED, and angiotensin-II has been implicated (John and Schmieder 2003; Akomolafe et al. 2016). Therefore, prevention of angiotensin-II production by the inhibition of ACE could be a therapeutic target in the management of hypertension and hypertensive-induced ED. Inhibition of ACE has also been reported to activate the release of NO and bradykinin; an active biomolecule in erectile function process (Oboh et al. 2015; Akomolafe et al. 2016). The inhibition of ACE activity in this study could be a function of interaction between the phenolics in the peels with disulphide bridge of the enzyme and/or chelation of zinc atom within the active site of the enzyme (John and Schmieder 2003; Ademiluyi et al. 2015). This is in line with our previous studies that phenolic-rich extracts from some plant inhibited ACE activity (Oboh et al. 2015; Ademiluyi et al. 2015; Akomolafe et al., 2016), Moreover, flavonoids such as rutin, quercetin and quercitrin which are dominant in UPP have been reported to be potent inhibitors of ACE activity (Guerrero et al. 2012; Shodehinde et al. 2016), and could be responsible for the higher inhibitory effect observed by UPP.

Oxidative damage of vital molecules such as polyunsaturated fatty acids, proteins and genetic materials by ROS and transition metals via peroxidation is of significant physiological importance in the pathophysiology of ED (Oboh et al. 2015; Akomolafe et al. 2016), as these leads to adverse effects such as inactivation of membrane enzymes and receptors, impairment of membrane permeability and apoptosis of cavernosal smooth muscle cells which could lead to reduced level of NO and endothelial dysfunction, and production of MDA (Khan et al. 2001; Agarwal et al. 2006). Hence, improving body antioxidant status could be helpful in combating oxidative damage, and one practical way is to improve/increase the consumption of phenolic-rich foods, as studies have shown in both *in vitro* and *in vivo* experimental models that the antioxidant property of plant foods is a function of their phenolics content (Shodehinde et al. 2015; Adefegha et al. 2015). Furthermore, the reducing power (ability to reduce Fe^3+^ to Fe^2+^) of the extracts is a function of their antioxidants capacity, which indicates their potential to prevent the production of oxidized intermediates of lipid peroxidation possibly via electron donation (Adefegha et al. 2015). Nevertheless, it is worth mentioning that UPP had higher antioxidant properties compared with RPP, and this could be associated with its high phenolic content/constituents. Our findings corroborate with earlier studies that have established a correlation between the phenolic contents and antioxidant properties of plant foods. The antioxidative potential of the peels could be explored as one of the mechanisms underlying its use in the management of ED.

Phenolics are prominent class of phytochemicals due to their ability to donate electrons and terminate the chain reaction process as a result of their (poly) hydroxyl groups, most especially the 3′OH and 4′OH of their three-carbon chain (Adefegha et al. 2015). These distinguishing structural features confer antioxidant and pharmacological importance on them (Agarwal et al. 2006; Adefegha et al. 2015). Several reports have shown that phenolic components possess PDE-5, arginase, AChE and ACE inhibitory potentials which could be linked to the formation of hydrogen bond and hydrophobic interactions between these polyphenolic compounds and the hydrophobic active site of the enzymes (Dos-Reis et al. 2013; Oboh et al. 2015; Akomolafe et al. 2016), and this could be responsible for the biological effects exhibited by the studied plantain peel extracts.

## Conclusions

The abilities of the UPP and RPP to inhibit crucial enzymes (PDE-5, arginase, AChE and ACE) and Fe^2+^ induced lipid peroxidation relevant to the management of ED could be due to their phenolic contents/constituents. These could, therefore, be possible mechanisms for the use of plantain peels in the management ED. Nevertheless, UPP extract appeared to be the most potent and may serve as source of nutraceticals in the treatment of ED. However, further *in vivo* and clinical studies are recommended.
